# Prior oxygenation, but not chemoreflex responsiveness, determines breath‐hold duration during voluntary apnea

**DOI:** 10.14814/phy2.14664

**Published:** 2021-01-04

**Authors:** Christina D. Bruce, Emily R. Vanden Berg, Jamie R. Pfoh, Craig D. Steinback, Trevor A. Day

**Affiliations:** ^1^ Department of Biology Faculty of Science and Technology Mount Royal University Calgary AB Canada; ^2^ School of Health and Exercise Sciences Centre for Heart, Lung and Vascular Health Faculty of Health and Social Development University of British Columbia Kelowna BC Canada; ^3^ Department of Biology Faculty of Science University of Victoria Victoria BC Canada; ^4^ Faculty of Kinesiology, Sport, and Recreation University of Alberta Edmonton AB Canada

**Keywords:** breath‐hold duration, hypoxic ventilatory response, oxygen, peripheral respiratory chemoreflex, respiratory chemoreceptors, steady‐state chemoreflex drive

## Abstract

Central and peripheral respiratory chemoreceptors are stimulated during voluntary breath holding due to chemostimuli (i.e., hypoxia and hypercapnia) accumulating at the metabolic rate. We hypothesized that voluntary breath‐hold duration (BHD) would be (a) positively related to the initial pressure of inspired oxygen prior to breath holding, and (b) negatively correlated with respiratory chemoreflex responsiveness. In 16 healthy participants, voluntary breath holds were performed under three conditions: hyperoxia (following five normal tidal breaths of 100% O_2_), normoxia (breathing room air), and hypoxia (following ~30‐min of 13.5%–14% inspired O_2_). In addition, the hypoxic ventilatory response (HVR) was tested and steady‐state chemoreflex drive (SS‐CD) was calculated in room air and during steady‐state hypoxia. We found that (a) voluntary BHD was positively related to initial oxygen status in a dose‐dependent fashion, (b) the HVR was not correlated with BHD in any oxygen condition, and (c) SS‐CD magnitude was not correlated with BHD in normoxia or hypoxia. Although chemoreceptors are likely stimulated during breath holding, they appear to contribute less to BHD compared to other factors such as volitional drive or lung volume.

## Graphical abstract

Breath holding stimulates respiratory chemoreceptors, likely affecting volitional breath‐hold duration (BHD). We tested the effects of varying prior oxygenation (PO_2_; hypoxia [~14% inspired], normoxia [room air], and hyperoxia [100% inspired]) and chemoreflex responsiveness on voluntary BHD. BHD was longer in the hyperoxic condition and shorter in the hypoxic condition compared to normoxia, but chemoreflex responsive did not determine BHD. A multitude of factors likely determine when breathing resumes following a voluntary breath hold.

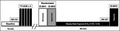

## 1 INTRODUCTION

Chemoreflexes play an important role in the respiratory control system by changing ventilation in response to fluctuations in arterial blood gases located at the bifurcation of the common carotid arteries, and the peripheral chemoreceptors (i.e., carotid bodies) detect changes in arterial O_2_ (PaO_2_) and CO_2_ (PaCO_2_). The peripheral chemoreflex (PCR) increases ventilation in response to rapid decreases in PaO_2_ (hypoxemia) and increases in PaCO_2_ (hypercapnia) in a synergistic fashion (Fitzgerald & Parks, 1971; Lahiri & DeLaney, 1975; López‐Barneo et al., 2016). The PCR plays a critical role in keeping arterial blood gases within normal ranges, particularly in the face of acute or chronic blood gas challenges, such as ventilatory acclimatization in hypobaric hypoxia (Mathew et al., 1983; Severinghaus et al., 1966). In other populations (e.g., chronic heart failure), increased PCR sensitivity may contribute to various disease states (Trembach & Zabolotskikh, 2017). Therefore, assessing PCR sensitivity may be relevant for clinical populations (e.g., sleep apnea) and healthy populations exposed to acute, chronic, or intermittent bouts of hypoxia (e.g., high altitude trekkers, breath‐hold divers).

Common methods used to indirectly assess PCR sensitivity in humans include eliciting transient, respiratory gas tests in order to measure the resulting ventilatory responses to assess chemoreflex magnitude (e.g., Pfoh et al., 2016; 2017). The short temporal nature of these tests allow investigators to assess respiratory responses independent of cardiovascular responses as well as ventilatory changes elicited from central chemoreceptor (CCR) stimulation (Pfoh et al., 2016). The hypoxic ventilatory response (HVR) test is one method used to assess PCR sensitivity to changes in oxygen. The HVR test can be elicited with acute, transient reductions in the fraction of inspired oxygen (i.e., F_I_O_2_) during poikilo‐ or isocapnic conditions (Nielsen & Smith, 1952; Pedersen et al., 1999; Steinback et al., 2007).

However, the methodology and nature of the HVR test pose limitations when applied to fieldwork or a clinical setting, requiring cumbersome equipment and potentially provoking respiratory discomfort. Recently, Pfoh et al. (2017) proposed a new method to assess steady‐state chemoreflex drive (SS‐CD). The SS‐CD provides an indirect measurement of the ventilatory strategy employed from contributions of both peripheral and CCRs in the steady state, given the prevailing chemostimuli, including pressure of end‐tidal (*P*_ET_)CO_2_ (Torr) and peripheral oxygen saturation (SpO_2_; %). Calculating the SS‐CD requires only steady‐state measures and minimal equipment, which could provide utility in fieldwork or clinical studies looking to assess chemosensitivity in the context of ventilatory acclimatization (e.g., Bruce et al., 2018). Voluntary breath‐hold duration (BHD) has also been explored as an alternative test for assessing peripheral chemoreceptor sensitivity (e.g., Feiner et al., 1995; Trembach & Zabolotskikh, 2017).

The act of voluntary breath holding (apnea) stimulates the PCR resulting from concomitant hypoxia and hypercapnia developing at the metabolic rate following the voluntary cessation of breathing. In the untrained person, BHD is determined by a multitude of factors including (a) initial blood gas concentrations (e.g., oxygen and carbon dioxide), (b) initial lung volume (e.g., afferent feedback from lung stretch receptors), (c) metabolic rate, and (d) volitional drive (Skow et al., 2015; see Parkes, 2006 for review). These factors are difficult to isolate in humans, but duration of a voluntary apnea is the most objective measure to use when assessing factors that contributed to the termination of a voluntary apnea in the untrained individual (i.e., break point; Lin et al., 1974). Two specific break points have been identified during a voluntary breath hold (Lin et al., 1974): the physiological and psychological break point. Physiological break point is identified as the onset of involuntary diaphragmatic contractions while the psychological break point is when volitional breath holding ends and breathing resumes. Physiological break point in individuals with no previous breath‐hold training will most often resume ventilation (psychological break point) from the unfamiliar sensation of an involuntary diaphragmatic contraction.

Intuitively, increased PCR sensitivity would potentially reduce apneic durations, resulting in a negative relationship between chemosensitivity and BHD. In fact, carotid body resection (Davidson et al., 1974) and vagal and glossopharyngeal nerve blockade (Guz et al., 1966) have been found to prolong BHD. Using mathematical modeling, Goncharov et al. (2017) concluded that at least 70% of BHD is influenced by chemosensitivity in untrained humans. The HVR test has previously been shown to predict BHD via multiple linear regression (when accounting for lung volume), whereas the hypercapnic ventilatory response test (HCVR; in hyperoxia) was not a predictor of BHD (Feiner et al., 1995). However, Trembach and Zabolotskikh (2017) found that the duration of an end‐inspiratory voluntary BH was strongly and negatively correlated with PCR CO_2_ sensitivity when quantified by a single‐breath CO_2_ test.

To what extent chemoreceptor sensitivity, when assessed via transient gas perturbation tests, predicts BHD in humans remains somewhat inconsistent in the literature, likely due to variability in quantifying chemoreceptor sensitivity and the constraints and confounds of testing neural control of breathing in vivo (Pfoh et al., 2016). In addition, the extent that SS‐CD may be related to breath holding is currently unknown. We aimed to characterize the effects of prior oxygenation, HVR, and SS‐CD magnitude on voluntary BHD under conditions of steady‐state hypoxia (13.5%–14% O_2_), normoxia (i.e., room air), and hyperoxia (five consecutive breaths of 100% O_2_) in order to assess the role of peripheral respiratory chemoreceptor activation on voluntary BHD. We hypothesized that (a) voluntary BHD would be positively related to the initial oxygen status (i.e., hypoxia, normoxia, or hyperoxia) prior to breath holding; (b) HVR responsiveness would be negatively related to BHD in hypoxia, normoxia, and hyperoxia; and (c) SS‐CD magnitude in both normoxia and hypoxia would be negatively correlated with voluntary BHD.

## 2 MATERIALS AND METHODS

### 2.1 Ethical approval

This study abided by the Canadian Government Tri‐Council policy on research ethics with human participants (TCPS2) and conformed with the standards set by the latest revision of the *Declaration of Helsinki*, except for registration in a database. Ethical approval was received in advance through the Mount Royal University Human Research Ethics Board (Protocol 2015‐26a). Following recruitment, all participants provided voluntary, informed, and written consent to the study prior to the beginning of the protocol.

### 2.2 Participant recruitment and inclusion criteria

**Figure 1:**
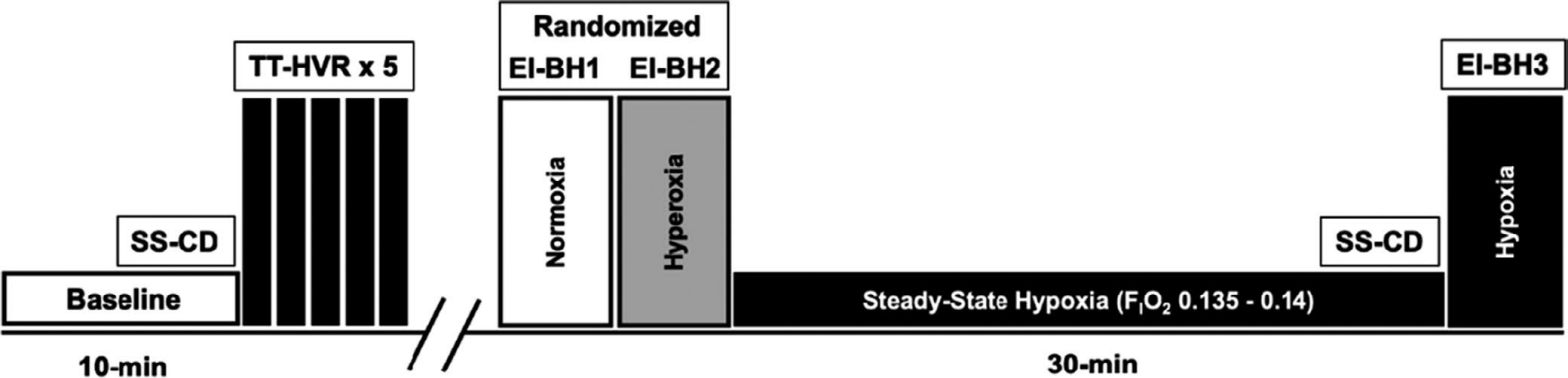
Protocol schematic. Following instrumentation, participants breathed room air for 10 min. We then carried out five consecutive transient hypoxic ventilatory response tests (TT‐HVR), comprising three consecutive normal tidal breaths of 100% N_2_. Following a return to baseline values, participants carried out two voluntary end‐inspiratory breath holds (EI‐BH), the first under room air conditions, and the second following five tidal breaths of 100% O_2_. Participants then breathed F_I_O_2_ 0.13.5–014 (13.5%–14%) for ~30 min to each steady state, after which they performed a third EI‐BH in hypoxic conditions

A total of 16 participants volunteered for the study (29.9 ± 8.0 years; BMI 23.9 ± 3.5 kg/m^2^; six males), with only a subset (*n* = 12) completing the transient HVR test (see Section 2.4, below). Participants were all non‐hypertensive with no reported history of neurological, cardiovascular, or respiratory illness and were not taking any prescription medications aside from hormonal birth control. Because the monthly fluctuation of cycling ovarian hormones (Macnutt et al., 2012) and gender (Pfoh et al., 2016) have been previously shown to be not related to HVR magnitude, no regard was given to the position in the ovarian cycle in the recruitment of women in this study. All participants were non‐smokers and abstained from caffeine, alcohol, and exercise for at least 12 hr prior to participation. Following pre‐screening and written informed consent, participants were familiarized with the protocol prior to instrumentation (Figure 1). All data were collected in a quiet and darkly lit laboratory during mid‐day. A subset of these participants were also recruited for a previously published study (Pfoh et al., 2017), and some HVR and SS‐CD values are overlapping in both studies. However, the question of this study was determined a priori in advance and is independent from Pfoh et al. (2017), which compared various chemoreflex tests.

### 2.3 Instrumentation and data collection

All data were collected using a 16‐channel PowerLab system (Powerlab/16SP ML880; AD Instruments; ADI) and analyzed offline using commercially available software (LabChart Pro software 8). Respiratory flow was measured through the use of a pneumotachometer (HR 800L flow head and spirometer amplifier; ADI ML141). Instantaneous ventilation (V̇_I_, L/min) was determined as the product of breath‐by‐breath inspired volume (*V*_TI_; calculated from the integral of the flow signal) and respiratory rate (*R*_R_, min^−1^; calculated by 60 per period of the flow signal). Breath‐by‐breath O_2_ and CO_2_ were sampled proximal to the mouth and measured in percent using a combined CO_2_ and O_2_ gas analyzer (ADI ML206), calibrated daily. The pressure of end‐tidal (*P*_ET_)O_2_ and *P*_ET_CO_2_ was calculated and corrected for BTPS in Torr using the daily atmospheric pressure (Calgary is ≈1,045 m with *P*_ATM_ of ≈665 mmHg). Calculating oxygen saturation (ScO_2_; (%)) was performed using the previously described equation (Severinghaus, 1979) as in previous studies in our laboratory (Pfoh et al., 2016, 2017) and measured by a peripheral pulse oximeter (SpO_2_, ADI ML320) placed on the index finger of the right hand. ScO_2_ and SpO_2_ were observed at all times to ensure values never fell below 70% saturation for safety. For cardiovascular variables, participants were instrumented for measuring heart rate (HR; ECG electrodes in lead II configuration using the ADI bioamp ML132) and beat‐by‐beat blood pressure (Finometer Pro, Finapres Medical Systems; calibrated for every subject). Instantaneous HR was calculated from the R–R interval (60 per period). Mean arterial pressure (MAP) was calculated as a mean from the raw finger photoplethysmography arterial pressure tracing. Participants kept their eyes closed throughout the protocol while listening to white noise via ear buds to minimize distraction, except when instructions on breath holding were provided.

### 2.4 Hypoxic Ventilatory Response

Following 10 min of quiet sitting for baseline measurements (Table 1), the HVR test was carried out (see Pfoh et al., 2016, 2017 for detailed methodology and background). The transient HVR test consisted of three consecutive inspired tidal breaths of 100% N_2_ from a 50 L Douglas bag. Expired gases were directed to room air once passing by the gas analyzer port proximal the mouthpiece of the participant. The transient HVR test was repeated five times, each separated by 1–2 min to ensure respiratory gases (*P*_ET_CO_2_ and *P*_ET_O_2_) and SpO_2_ levels returned to baseline. To administer the transient test, an investigator manually switched the three‐way valve between room air and the 100% N_2_ gas mixture while monitoring the participants’ respiratory flow signal. Switching between the inspired gas mixtures was performed during expiration, largely without the participants’ awareness.

**Table 1:** Breath‐hold duration and baseline variables in room air and in steady‐state normobaric hypoxia

Variable	Room air (21% O_2_)	Hypoxia (13.5%–14% O_2_)	Hyperoxia (5 × 100% O_2_)
EI‐BHD (s)	53.8 ± 16.2**	40.4 ± 13.8**	89.9 ± 38.2**
HR (min^−1^)	66.7 ± 15.3	73.1 ± 18.0*	—
MAP (mm Hg)	102.1 ± 7.9	99.5 ± 7.3	—
R_R_ (min^−1^)	11.3 ± 3.3	12.6 ± 4.0	—
*V*_TI_ (L)	1.03 ± 0.4	1.1 ± 0.2	—
*V̇*_I_ (L/min)	10.6 ± 1.5	11.9 ± 1.9*	—
*P*_ET_CO_2_ (Torr; BTPS)	33.6 ± 2.1	31.7 ± 1.8*	—
*P*_ET_O_2_ (Torr; BTPS)	85.0 ± 3.6	43.3 ± 3.5*	—
ScO_2_ (%)	96.4 ± 0.4	78.6 ± 3.6*	—
SpO_2_ (%)	96.9 ± 0.9	78.4 ± 4.0*	—
SS‐CD (V̇_I_/SI; a.u.)	30.7 ± 5.2	29.3 ± 4.3	—
HVR (∆V̇_I_/∆ScO_2_; L/min/%)	0.38 ± 0.18	—	—

Note

Baseline data obtained while breathing room air and after reaching steady state while breathing 13.5%–14% F_I_O_2_ (~30‐min).

Abbreviations: and HVR, hypoxic ventilatory response (tested via 5× transient N_2_ test). EI‐BHD, end‐inspiratory breath‐hold duration; HR, heart rate; MAP, mean arterial pressure; *P*_ET_CO_2_, partial pressure end‐tidal carbon dioxide; *P*_ET_O_2_, partial pressure end‐tidal oxygen; *R*_R_, respiratory rate; ScO_2_, calculated oxygen saturation; SpO_2_, peripheral oxygen saturation by pulse oximetry; SS‐CD, steady‐state chemoreflex drive; *V̇*_I_, ventilation; *V*_TI_, inspired tidal volume.

EI‐BHD in hyperoxia (third column) is following five consecutive tidal breaths of 100% F_I_O_2_. Values are mean ± *SD*.

* Statistically different than room air (*p* < .05);

** Statistically different than all other conditions (*p* < .001).

### 2.5 Breath holding and steady‐state hypoxia

Three separate breath holds were performed following the HVR protocol. Participants were first coached through two, randomized, maximal breath‐hold maneuvers initiated at the end of a normal inspiration: one following breathing room air (normoxia), and another following breathing five normal tidal breaths of 100% F_I_O_2_ from a 50 L Douglas bag (i.e., hyperoxia). For these two randomized maneuvers, breath holds were initiated when all variables returned to baseline breathing room air (~5–10 min). Following a 10‐min break, the participant was then exposed to a F_I_O_2_ of approximately 0.135–0.14 (13.5%–14%), equating to simulated 4,500–5,000m of altitude. Once the participant reached steady state (i.e., unchanging variables; e.g., ventilation, *P*_ET_CO_2_, and SpO_2_; ~30 min), they performed the third breath‐hold maneuver. The initiation of each breath hold began with five coached breaths at their normal (i.e., resting) tidal volume to avoid anticipatory changes in breathing rate or tidal volume (leading to hypocapnia), and each breath hold was held until volitional break point. No practice breath holds were performed, nor were participants given encouragement throughout the breath hold. Each participant completed testing during a single laboratory visit (~2 hr).

### 2.6 Data Analysis and Statistics

#### 2.6.1 Baseline Data

For all baseline measures, a mean value over 30 s (i.e., 30‐s bin) that appeared most stable and representative for each participant's baseline was calculated from a 1‐min section at least 30‐s prior to the first HVR test. For cardiovascular variables, we quantified beat‐by‐beat HR (min^‐1^) and MAP (mm Hg). For respiratory variables, we quantified respiratory rate (*R*_R_; min^−1^), breath‐by‐breath inspired volume (*V*_TI_; L), inspired ventilation (V̇_I_, L/min; the product of breath‐by‐breath V_T_ and *R*_R_), *P*_ET_CO_2_ and *P*_ET_O_2_ (Torr; BTPS), and ScO_2_ (%; calculated from the equation described by Severinghaus, 1979). ScO_2_ was also used to avoid the well‐described delay in measuring peripheral pulse oximetry (Trivedi et al., 1997) and the known differences between SpO_2_ and ScO_2_ in hypoxic conditions (Pfoh & Day, 2016; Pfoh et al., 2016, 2017). We estimated V̇CO_2_ from *V̇*_I_ and peak fraction of expired (*F*_E_)CO_2_ values in the steady state in both normoxia and hypoxia (*V̇*_I_ × F_E_CO_2_/2), corrected for STPD (0.813 conversion factor).

#### 2.6.2 Hypoxic Ventilatory Response

For each trial, ventilatory responses were calculated as a change in ventilation from baseline values to the peak responses (Δ*V̇*_I_) over a change in stimulus (ΔScO_2_). The ΔV̇_I_ was calculated as the difference (delta) between baseline V̇_I_ prior to the test (15 s bin) and as the average *V̇*_I_ of the two largest consecutive breaths within the first 20 s following the stimulus, wherever it occurred, similar to previous studies in our laboratory (Pfoh et al., 2016; 2017). The ScO_2_ stimulus was calculated using the visually identified *P*_ET_O_2_ on the third 100% N_2_ breath. The average response of five trials was taken as the representative value for that participant.

#### 2.6.3 Breath Holding

Using respiratory flow in conjunction with inspired and expired volume measurements, BHD was determined using LabChart. Although we did not attempt to measure the physiological breakpoint (e.g., respiratory effort belt), with participants being untrained in breath holding, their final break point most likely represented both psychological and physiological break point (Parkes, 2006).

#### 2.6.4 Calculating SS‐CD

During baseline periods while breathing room air and during steady‐state hypoxia, a stimulus‐index was calculated (SI; P_ET_CO_2_/SpO_2_; Torr/%; e.g., Bruce et al., 2016; Pfoh et al., 2017) to represent the contributions from CO_2_ and O_2_ chemostimuli acting on both central and peripheral chemoreceptors (i.e., P_ET_CO_2_ and SpO_2_). The SI was then indexed against baseline V̇_I_ during both normoxic and hypoxic steady‐state conditions to calculate SS‐CD (Pfoh et al., 2017; see Table 1).

### 2.7 Statistics

All values are reported in Table 1 and Results as mean ± standard deviation (*SD*). Statistical significance was assumed when *p* < .05 (SigmaPlot v14, Systat).

Average baseline values during room air and steady‐state hypoxia are summarized in Table 1 for all participants. Paired *t*‐tests were used to test for differences in variables between normoxia and hypoxia.

**Figure 2:**
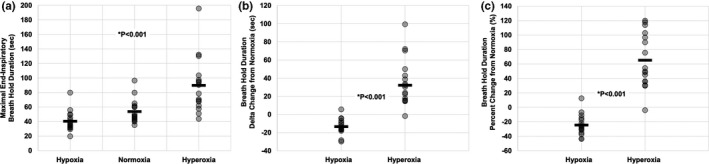
Breath‐hold duration (BHD) in steady‐state hypoxia, normoxia, and hyperoxia. (a) Absolute BHD in steady‐state hypoxia (13.5%–14% O_2_; 40.4 ± 13.8 s), normoxia (room air; mean 53.8 ± 16.2 s), and hyperoxia (following five tidal breaths of inspired 100% O_2_; 89.9 ± 38.2 s). (b) Changes in BHD from normoxia in steady‐state hypoxia (−13.4 ± 8.7 s) and hyperoxia (36.1 ± 26.1). (c) Percent changes in BHD from normoxia in steady‐state hypoxia (−24.5 ± 14.5%) and hyperoxia (65.1 ± 37.7%). Each transparent circle represents one individual, and horizontal bars represent the mean. *Significantly different from normoxic breath hold (*p* < .001)

In order to assess the statistical relationship between apneic duration and initial oxygen status, a one‐factor repeated‐measures analysis of variance (ANOVA) test was performed (Table 1 and Figure 2a). When significant *F*‐ratios were detected, a Student–Newman–Keuls *post hoc* test was used for multiple pair‐wise comparisons between the three oxygen levels (hypoxia, normoxia, and hyperoxia; Figure 2a).

Paired *t*‐tests were used to compare the delta (Figure 2b) and percent change (Figure 2c) in BHD from normoxia between hypoxia and hyperoxia.

**Figure 3:**
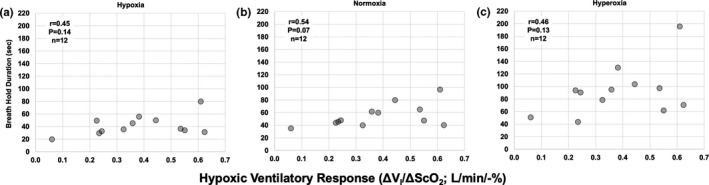
Within‐individual correlations between absolute BHD and the hypoxic ventilatory response (HVR). (a) Relationship between Absolute BHD in steady‐state hypoxia (13.5%–14% O_2_) and HVR (*r* = .45, *p* = .14, *n* = 12). (b) Relationship between absolute BHD in normoxia (i.e., room air) and HVR (*r* = .54, *p* = .07, *n* = 12). (c) Relationship between Absolute BHD in hyperoxia (following five breaths of 100% O_2_) and HVR (*r* = .46, *p* = .13, *n* = 12). The respective *r* value (Pearson correlation coefficient), *p* value, and *n* are presented on each graph

**Figure 4:**
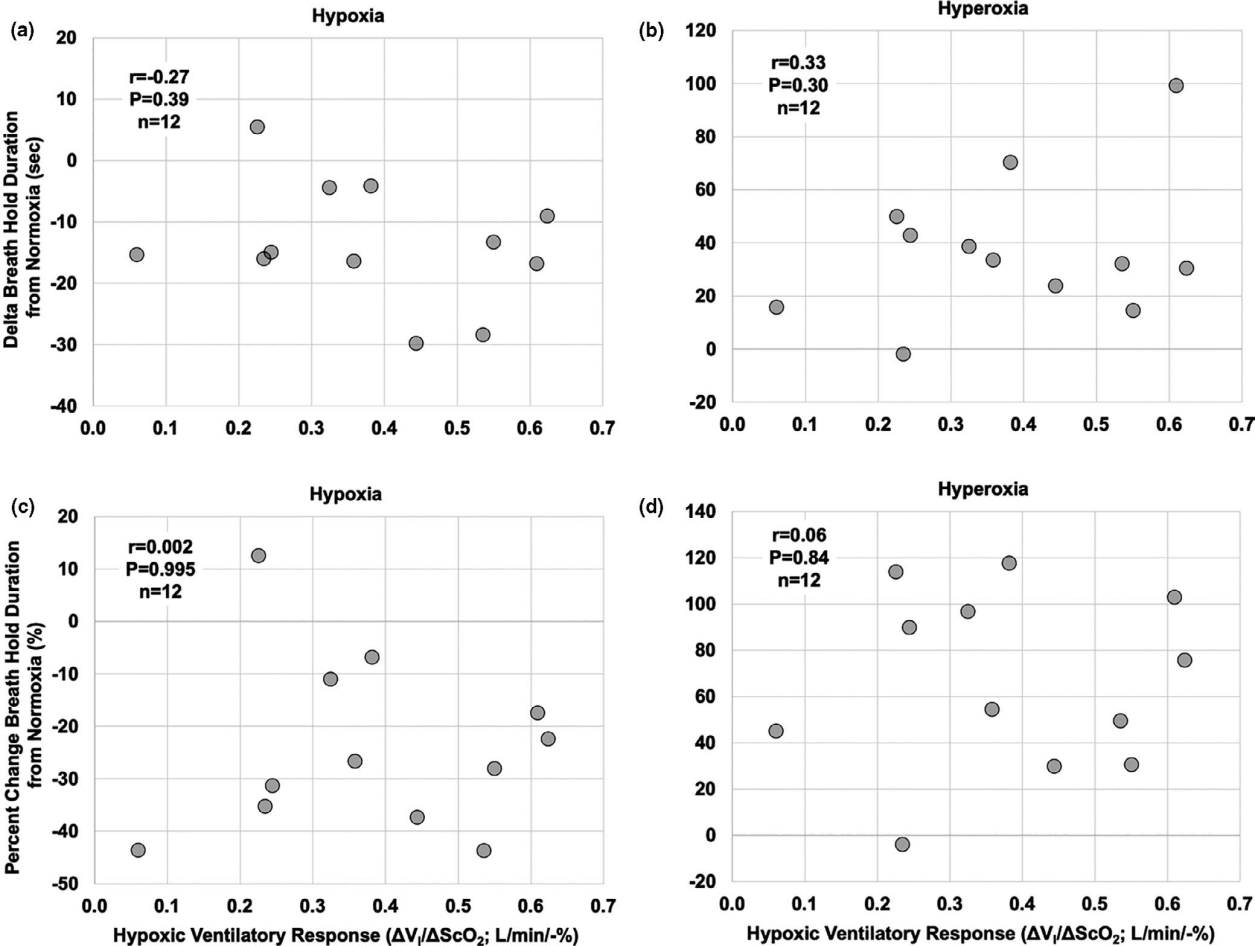
Within‐individual correlations between relative BHD and the hypoxic ventilatory response (HVR). (a) Relationship between delta BHD from normoxia in steady‐state hypoxia (13.5%–14% O_2_) and HVR (*r* = −.27, *p* = .39, *n* = 12). (b) Relationship between delta BHD from normoxia in hyperoxia (following five breaths of 100% O_2_) and HVR (*r* = .33, *p* = .3, *n* = 12). (c) Relationship between percent change in BHD from normoxia in steady‐state hypoxia and HVR (*r* = .002, *p* = .995, *n* = 12). (d) Relationship between percent change BHD from normoxia in hyperoxia and HVR (*r* = .06, *p* = .84, *n* = 12). The respective *r* value (Pearson correlation coefficient), *P* value, and n are presented on each graph

Pearson product moment correlation tests were used to assess relationships between (a) all absolute breath hold durations (BHD) in hypoxia, normoxia, and hyperoxia (Figure 3a–c) and the HVR; (b) the delta (Figure 4a,b) and percent change (Figure 4c,d in BHD from normoxia and the HVR in hypoxia (Figure 4a,c) and hyperoxia (Figure 4b,d); and (c) the relationship between BHD in normoxia and hypoxia and the respective SS‐CD.

Lastly, a paired *t*‐test was also used to compare SS‐CD between normoxia and hyperoxia.

## 3 RESULTS

### 3.1 Cardiorespiratory Variables in Normoxia versus Hypoxia

Table 1 presents baseline cardiorespiratory data while breathing room air (normoxia) and after reaching steady state (~30 min) while breathing steady‐state hypoxia (F_I_O_2_ 0.135–0.14). Of note, HR, *V̇*_I_, *P*_ET_CO_2_, *P*_ET_O_2_, ScO_2_, and SpO_2_ were all statistically different in hypoxia compared to normoxia. Estimated V̇CO_2_ was marginally, but significantly, higher in steady‐state hypoxia compared to room air (249.3 ± 46.5 versus 232.8 ± 32.8 ml/min, respectively; +7.0 ± 12.9% increase in hypoxia; *p* = .03).

### 3.2 Normoxic, hyperoxic, and hypoxic BHDs

For the normoxic breath hold (i.e., breathing room air), participants held their breath, on average for 53.8 ± 16.2 s. Hyperoxic BHDs were longer than normoxia at 89.9 ± 38.2 s (*p* < .001). Breath holds following steady‐state hypoxia were statistically shorter than normoxia at 40.4 ± 13.8 s (*p* < .001; see Figure 2a). In addition, compared to room air (i.e., normoxia), the steady‐state hypoxic breath hold was −13.4 ± 8.7 s (−24.5 ± 3.6%) shorter, while the hyperoxic breath hold was + 36.1 ± 26.1 s (+65.1 ± 37.7%) longer (Figure 2b,c).

### 3.3 HVR and SS‐CD

**Figure 5:**
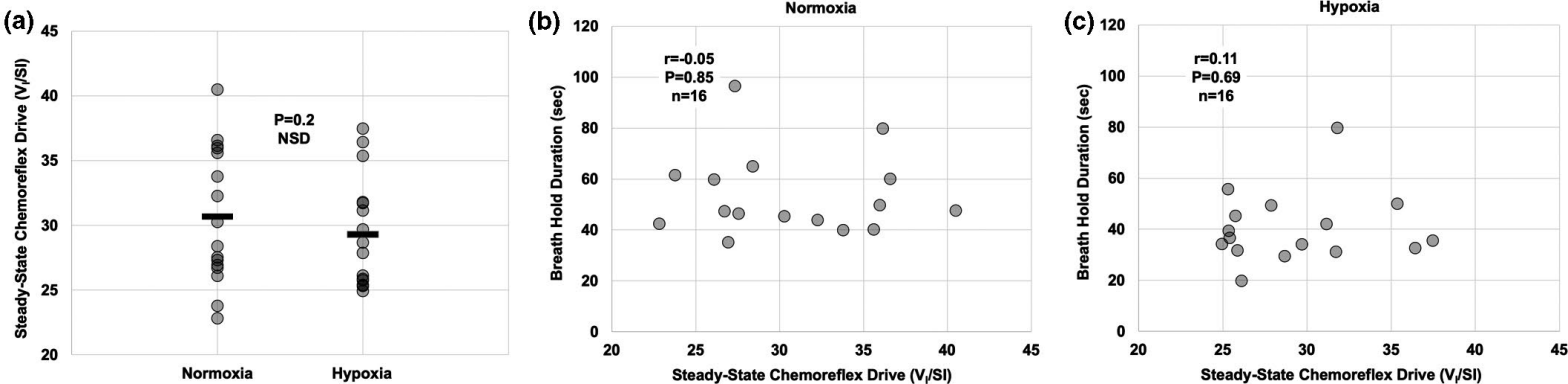
Within‐individual correlations between relative BHD and steady‐state chemoreflex drive (SS‐CD). (a) Comparison of SS‐CD in normoxia (room air) and hypoxia (13.5%–14% O_2_). (b) Correlation between absolute BHD and SS‐CD in normoxia (*r* = −.05, *p* = .85, *n* = 16). (c) Correlation between absolute BHD and SS‐CD in hypoxia (*r* = .11, *p* = .69, *n* = 16). NSD, no significant difference (*p* > .05). The respective *r* value (Pearson correlation coefficient), *p* value, and *n* are presented on each graph

Using the transient 100% N_2_ test, the HVR was on average 0.38 ± 0.18 L/min/% ScO_2_ (*n* = 12; Table 1), similar to previous reports in our laboratory (Pfoh et al., 2016, 2017). SS‐CD was not different between normoxia (30.7 ± 5.2) and hypoxia (29.3 ± 4.3; *p* = .2; *n* = 16; Figure 5a), similar to previous reports in our laboratory (Bruce et al., 2018; Pfoh et al., 2017).

### 3.4 BHD and the HVR

Both absolute (delta) and relative (% change from normoxia) BHDs appeared to have no significant relationship with HVR magnitude. BHD in hypoxia (Figure 3a), normoxia (Figure 3b), and hyperoxia (Figure 3c) was not related to the HVR (*r* < .54; *p* > .07, *n* = 12). The change in BHD from normoxia to hypoxia was not correlated with HVR (*r* = −.27, *p* = .39, *n* = 12; Figure 4a), or was the change in BHD from normoxia to hyperoxia (*r* = 0.33, *p* = .3 *n* = 12; Figure 4b). Similarly, percent change in BHD from normoxia to hypoxia was not correlated with HVR (*r* = .002, *p* = .995, *n* = 12; Figure 4c), or was the percent change in BHD from normoxia to hyperoxia (*r* = .06, *p* = .84, *n* = 12; Figure 4d).

### 3.5 BHD and SS‐CD

There were no differences in SS‐CD between normoxia and hypoxia (*p* = .2; Figure 5a). Voluntary BHD was not correlated with SS‐CD in either normoxia (*r* = −.05, *p* = .85, *n* = 16; Figure 5b) or hypoxia (*r* = .11, *p* = .69, *n* = 16; Figure 5c).

## 4 DISCUSSION

We aimed to assess the relationship between prior oxygenation on voluntary BHD, and to assess the possible relationship among BHD, HVR, and SS‐CD, within individuals. The principal findings of our study are as follows: (a) BHD duration was dependent on the initial fraction of inspired oxygen immediately before voluntary apnea, such that BHD is positively related to prior oxygenation in a dose‐dependent fashion (i.e., shorter in hypoxia and longer in hyperoxia); (b) hypoxic, normoxic, and hyperoxic BHD were not related to the HVR; and (c) normoxic and hypoxic BHD were not correlated with SS‐CD, which takes into account both central and peripheral chemoreceptor contributions to BHD in the steady state.

### 4.1 BHD and Prior Oxygenation

We found that alterations in prior F_I_O_2_ affected voluntary BHD, indicating peripheral chemoreceptor activation (hypoxia; i.e., decreased duration) and inhibition/withdrawal (hyperoxia; increased duration) plays an important role in determining BHD. These results are consistent with previous studies, which assessed the effects of initial oxygen status on voluntary BHD (e.g., Engel et al., 1946; Ferris et al., 1946; Godfrey & Campbell, 1969; Klocke & Rahn, 1959). For example, Engel et al. (1946) found inspired oxygen tensions (below that of sea level values) prior to initiating a breath‐hold drastically decreased duration, which was less pronounced when approached prior inspired oxygen tensions of 100%. Although our results do not suggest an exponential relationship *per* se between prior oxygen tension and BHD, our data still indicate BHD is somewhat dependant on initial inspired oxygen.

### 4.2 BHD and Chemoreflex Magnitude

Breath holding is a unique stressor, eliciting incremental hypoxia, hypercapnia, and sympathetic nervous system activation (Hagbarth & Vallbo, 1968; Lin et al., 1974; Steinback et al., 2009). These concomitant blood gas chemostimuli accumulate at the metabolic rate, stimulate both central and peripheral chemoreceptors, which continuously increases the drive to breathe and likely reduces BHD. Thus, chemoreceptor stimulation or withdrawal is contributing to the timing of break point during a voluntary breath hold. However, it is interesting that BHD did not correlate with HVR magnitude using a transient HVR test, which quantifies the PCR sensitivity to transient reduction in arterial hypoxia. Thus, although chemoreceptor stimulation influences BHD, chemoreflex magnitude does not appear to *determine* voluntary BHD. The lack of correlation between voluntary apnea duration and chemoreceptor sensitivity measured as HVR may be a result of (a) the known variability inherent in transient gas perturbation tests (Pfoh et al., 2016; 2017), (b) the prevailing CO_2_ at the beginning of and throughout the BH and the concomitant sensitivity of the carotid bodies to CO_2_ (e.g., Trembach & Zabolotskikh, 2017), and (c) the contribution of central respiratory chemoreceptors.

Few studies have assessed the relationship between chemoreflex magnitude and BHD. Bain et al. (2017) showed that forced vital capacity (an index of lung volume) was a good predictor of BHD following administration of hyperoxia in elite apneists, but that the central chemoreflex magnitude, assessed via hyperoxic rebreathing tests prior to the apnea, was not predictive of duration. Feiner et al. (1995) showed that the HVR was a significant predictor of BHD, but only when the effect of lung volume was included in statistical analysis. In addition, Feiner et al. (1995) also found no relationship between voluntary BHD and the central hypercapnic ventilatory response. Conversely, Trembach and Zabolotskikh (2017) found that PCR magnitude to CO_2_, tested via single breath test, was strongly, significantly, and inversely correlated with voluntary BHD in a large group of healthy participants. This is difficult to reconcile with the findings from our study, where the HVR not significantly correlated with BHD in normoxia or hypoxia, particularly given that we found previously that the PCR magnitude of HVR via transient N_2_ test was well correlated with the PCR magnitude tested via the single‐breath CO_2_ test, within individuals (Borle et al., 2017), which was also consistent with a previous study (Rebuck et al. (1973). Thus, an integrative model of the underlying physiological and extra‐physiological mechanisms determining volitional break point remains to be determined.

Because voluntary apnea elicits a simultaneous blood gas, circulatory and neural responses that increase cerebral perfusion (e.g., Steinback & Poulin, 2008; Steinback et al., 2009; Willie et al., 2012), while limiting perfusion of non‐vital organs (i.e., the mammalian diving reflex; Dujic & Breskovic, 2012; Dujic et al., 2013; Joulia et al., 2009; Lin, 1982), the CCRs may have played a smaller role in determining break point compared to the PCRs. This mechanism would theoretically dampen the CO_2_/H^+^ stimulus to the CCRs by increasing cerebral blood flow and, thus, promoting metabolic washout from cerebral tissues (e.g., Ainslie & Duffin, 2009; Bruce et al., 2016). Although cerebrovascular reactivity (CVR) likely dampens the role of CCR stimulation on determining BHD, this conclusion is only speculation without assessing a relationship between CCR and CVR during BHD.

### 4.3 Possible Effects of CO_2_/[H^+^]

The incremental increase in CO_2_ during breath holding likely stimulates the central respiratory chemoreceptors, increasing the drive to breathe and likely shortening BHD (Ferris et al., 1946). The concomitant hypercapnia during breath holding also plays a role in stimulating PCRs, particularly as oxygen levels decrease during apnea, given that the PCRs detect changes in oxygen in a multiplicative, CO_2_/[H^+^]‐dependent fashion (e.g., Fitzgerald & Parks, 1971; Kiwull‐Schone et al., 1976; Lahiri & DeLaney, 1975; Lahiri et al., 1978; Van Beek et al., 1983). In our study, these increases in CO_2_ may confound our results in two possible ways: (a) the metabolic rate (i.e., CO_2_ accumulation) may have been different during the three BH trials, (b) hypoxia‐induced lactic acid accumulation may alter hypoxic sensitivity, and (c) the baseline (i.e., prior to breath holding) *P*_ET_CO_2_ may have been different between conditions. The mean metabolic rate (i.e., V̇CO_2_) was only slightly (but significantly) higher (~7%) after 30‐min hypoxia, which likely played a limited role in increasing CO_2_ during breath holding under hypoxic conditions. In addition, 30 min of hypoxia may have generated increased blood lactate, contributing to arterial acidosis, which we did not measure. However, Ainslie et al. (2014) showed that following 15‐min steady‐state hypoxia at levels similar to our study (P_ET_O_2_ ~ 42 Torr, SaO_2_ ~ 78%), arterial lactate was not increased statistically, however, it was significantly elevated at more extreme hypoxia (*P*aO_2_ ~ 36 Torr, SaO_2_ ~ 70%; 0.6 to 0.7 mmol/L). With respect to more chronic hypoxia, Smith et al. (2014) showed that on days 4–6 at 5,050 m following 9 days of incremental ascent (PaO_2_ ~ 42 Torr, SaO_2_ ~ 81%), arterial lactate was only mildly but statistically elevated (0.7 at baseline to 0.9 mmol/L). Conversely, baseline P_ET_CO_2_ was lower in hypoxia by approximately 2 Torr (see Table 1), which was caused by and subsequently inhibited the HVR (i.e., poikilocapnic hypoxia; e.g., Steinback & Poulin, 2007; see Table 1). Indeed, ventilation was statistically higher at baseline after 30 min of hypoxia, but only by approximately 1 L/min on average. Thus, participants had higher ventilation in hypoxia, but it was blunted by the relative concomitant hypocapnia. These two antagonistic factors, where V̇CO_2_ was higher but starting CO_2_ was lower compared to the normoxic BH, (a) are likely negligible in their contribution to BHD and (b) likely cancel each other out across the breath hold, leaving the effects of prior oxygenation as the key variable driving differences in BHD in our study. Indeed, this complex interplay in generating a new steady state in the face of antagonistic stimuli was in part the initial justification for developing and applying our SS‐CD metric (see Bruce et al., 2018; Leacy et al., 2020; Pfoh et al., 2017).

### 4.4 BHD and SS‐CD

We developed a metric of SS‐CD, which encompasses the ventilatory strategy employed in response to the prevailing CO_2_ and O_2_ in the steady state (Pfoh et al., 2017). SS‐CD was not different between room air and steady‐state hypoxia, likely due to the fact that P_ET_CO_2_ and SpO_2_ both decreased a similar magnitude, while ventilation was only minimally increased (~1 L/min) compared to room air values once steady state was reached in hypoxia (see Table 1). Our results suggest that the SS‐CD does not determine BHD when compared in room air and normobaric steady‐state hypoxia. Although we found no correlations between BHD and SS‐CD magnitude in either acute normoxia or hypoxia, there may be merit in assessing the relationship between BHD and SS‐CD during chronic hypoxic conditions (i.e., high altitude), where hypobaric hypoxia decreases voluntary breath hold duration (e.g., Ferris et al., 1946) and ventilatory acclimatization increases SS‐CD (e.g., Bruce et al., 2018; Leacy et al., 2020). However, it is possible that the temporal domain of breath holding (i.e., acute dynamic chemostimulation) and calculating the SS‐CD (i.e., obtained during steady state) may be unrelated. In other words, ventilatory chemoresponses to dynamic and incremental changes in blood gases (both CO_2_ and O_2_) may be more relevant to driving voluntary BHD.

### 4.5 Potential Contribution of Extra‐Chemoreceptor Factors

Despite the clear contribution of initial oxygen status on voluntary BHD demonstrated by this and other studies, neither the transient HVR nor SS‐CD was significantly correlated with BHD. Thus, despite stimulation of central and peripheral respiratory chemoreceptors before and during the breath hold, chemoreflex magnitude does not appear to determine voluntary BHD, implicating extra‐chemoreceptor factors. Previous studies demonstrated that sensitivity of the carotid body is not solely responsible for apnea break point, as individuals following carotid body denervation still voluntarily terminated a breath hold (Davidson et al., 1974). In one of the most striking examples of extra‐chemoreceptor contributions, Campbell et al. (1966), Campbell et al. (1967), Campbell et al. (1969), showed that voluntary BHD was increased by two to threefold in two participants following full paralyzation (aside from an upper arm), where participants indicated their volitional break point with a hand signal from the unanesthetized hand. These authors suggested that a portion of the distressing sensations elicited by breath holding arise through the elimination of respiratory muscle contraction and related inhibitory afferent feedback on the drive to breath (Campbell et al., 1967). Indeed, Fowler, 1954; later confirmed by Flume et al., 1994) showed that if participants rebreathed from a bag containing hypoxic and hypercapnic air at break point, they could continue breath holding, despite no change in blood gases, illustrating that the act of breathing itself relieves the symptoms of distress associated with breath holding, at least initially. However, as the successive break point–rebreathing cycle continues, the BHD shortens, illustrating a role for chemoreceptor stimulation in the sensation of breathlessness and voluntary BHD.

In addition to prior oxygenation and afferent feedback from contracting respiratory muscles, other factors contribute to BHD, such as the initial lung volume (e.g., Feiner et al., 1995; Godfrey & Campbell, 1968; 1969; Guz et al., 1966) and volitional factors (Parkes, 2006). In our study, we coached participants to initiate the breath hold at the end of a normal inspiration, where lung volume at the onset of each apnea was not equivalent between conditions (e.g., smaller tidal volumes during normoxia compared to hypoxia; see Table 1). However, given the difference between measured tidal volume was ~ 100ml between normoxia and hypoxia, these differences likely contributed little to BHD in comparison to the measured differences in F_I_O_2_. Lastly, performing an additional motor or cognitive task (Alpher et al., 1986) as well as the simple act of training (Engan et al., 2013) can prolong BHD. The potential effects of training and volition were controlled for in our study by selecting untrained participants, and providing no encouragement to the participants throughout their apnea. Unfortunately, the effect of training somewhat difficult to control for when multiple breath holds are required for data collection, as participants can often breath hold for longer durations with subsequent apneas in the same session (unpublished observations). Taken together, these considerations demonstrate that breathing holding is a complex physiological stressor, with multiple factors contribution to the ability of participants to voluntarily resist one of our most primitive and immediate physiological drives (e.g., Parkes, 2006).

### 4.6 Possible Relationship to Sleep Apnea

Central and obstructive sleep apnea are comprised of repeated cycles with alternating phases of breath holding and hyperventilation during sleep (Douglas et al., 1982; Sin et al., 2015). Previous experimental and modeling studies demonstrated a relationship between chemoreflex responsiveness (i.e., gain) and the severity of central sleep apnea, particularly with ventilatory acclimatization to high altitude (Ainslie et al., 2013). Because of the differences in temporal delay in stimulating peripheral (short) and central (long) chemoreceptors (e.g., Pederson et al., 1999), it is likely that peripheral chemoreceptors play a more important role in determining the apnea–hyperventilation cycle lengths. In addition, the transient chemoreflex tests utilized to assess HVR or HCVR are similar to the kind of chemostimuation sleep apnea patients’ experience (i.e., short duration chemostimulation). However, perhaps combining transient hypoxia and hypercapnia into a single transient test may be a more relevant “real world” stimulus. Conversely, although a strength of the SS‐CD metric is that it takes into account the ventilatory strategy employed in response to the prevailing CO_2_ and O_2_ in the steady state, that this metric is not in fact a measure of gain (responsiveness) to a perturbation in the way the hyperventilation following an apneic event is, may explain the lack of a relationship between SS‐CD magnitude and BHD in either normoxia and hypoxia. Using the appropriate test to assess chemoreflex gain when assessing its relationship to the severity of sleep apnea remains an important and evolving question (e.g., Messineo et al., 2018; Solin et al., 2000).

## 5 CONCLUSION

We assessed the relationship between varying levels of inspired oxygen and voluntary BHD in a laboratory setting, and assessed the possible relationships between both prior inspired oxygen (i.e., hypoxia, normoxia, and hyperoxia) and SS‐CD on voluntary BHD. We found that BHD was positively related to prior oxygen status, shorter in hypoxia and longer in hyperoxia. However, BHD was not related to (a) transient HVR or (b) SS‐CD, a metric combining all chemostimuli in the steady state. We conclude that voluntary BHD is oxygen dependent, likely through PCR activation, but that extra‐chemoreflex factors determine BHD in untrained individuals, such as lung volume, lung stretch, and volition.

## CONFLICT OF INTEREST

None declared.

## AUTHOR CONTRIBUTIONS

TAD, ethics, funding, and laboratory where all experiments took place, conception and design of the work; TAD, JRP, CDB, EV, and CDS, data acquisition, analysis, and/or interpretation of data for the work; All co‐authors were involved in drafting and/or critically revising the work for important intellectual content.

## FUNDING INFORMATION

Financial support for this work was provided by (a) an NSERC USRA (C.D.B.), (b) an MRU Internal Research Grants fund (J.R.P.), (c) an MRU Distinguished Faculty Award fund (J.R.P.), (d) a University of Victoria Co‐op (E.R.V.), and (e) a Natural Science and Engineering Research Council of Canada Discovery grant (TAD; RGPIN‐2016‐04915).
